# Dataset on seed details of wheat genotypes, solution treatments to measure seedling emergence force and the relation between seedling force and strain

**DOI:** 10.1016/j.dib.2018.10.134

**Published:** 2018-10-30

**Authors:** Monia Anzooman, Yash P. Dang, Jack Christopher, Michael H. Mumford, Neal W. Menzies, Peter M. Kopittke

**Affiliations:** a The University of Queensland, School of Agriculture and Food Sciences, St Lucia, Queensland 4072, Australia; b The University of Queensland, Queensland Alliance for Agricultural and Food Innovation, Leslie Research Facility, Toowoomba, Queensland 4350, Australia; c Department of Agriculture and Fisheries, Queensland, Leslie Research Facility, Toowoomba, Queensland 4350, Australia

## Abstract

The seed details (weight, vigor) and germination rate of 16 wheat (*Triticum aestivum*) genotypes in a non-limiting conditions were measured. The dataset presents seed germination rate and seed vigor of 16 wheat genotypes. The dataset also presents the concentrations of the cations to create solution treatments of various sodium adsorption ratio (SAR) and ionic strength (*I*). Finally, dataset presented a figure of the experimental design to measure seedling emergence force of wheat genotypes. The image of the setup and the relation between strain and force have been presented here to convert the strain of the beam into seedling emergence force. This dataset has been used in research work titled ‘Greater emergence force and hypocotyl cross sectional area may improve wheat seedling emergence in sodic conditions’ (Anzooman et al., 2018) [1].

**Specifications table**

**Table:** 

Subject area	*Physics, Chemistry*
More specific subject area	Solution concentration, strain and force
Type of data	Table, image, graph, figure
How data was acquired	Microscope and manual measurement. Data logger (Quantum X data acquisition system DA, MX840B, HBM, Germany), slide calipers
Data format	Raw, filtered and analyzed
Experimental factors	Seeds were harvested in November 2016 and were stored in a cold room at 7 °C, and a week prior to the present experiment, seeds were warmed to 22 °C.
Experimental features	For each of 16 wheat (*Triticum aestivum)* varieties, the thousand-grain weight (TGW), the length and width were measured. Using digital slide calipers, the length and width of the seeds were measured for 50 replicate seeds of each genotype. We created a total of 16 treatment solutions, consisting of four ionic strength (*I)* values four sodium adsorption ratios (SARs) to measure seedling emergence force. Finally we measured the seedling emergence force using a mechanical device and developed a calibration curve to convert the strain into seedling emergence force.
Data source location	Seeds were collected from a harvest site at the Queensland Government research farm in Kingsthorpe, Queensland, Australia (27.52 °S, 151.79 °E).
Data accessibility	Data is with this article
Related research article	Anzooman, M, Dang, YP, Christopher, J, Mumford, MH, Menzies, NW & Kopittke, PM 2018, ׳Greater emergence force and hypocotyl cross sectional area may improve wheat seedling emergence in sodic conditions׳, Plant Science. doi:10.1016/j.plantsci.2018.09.007

**Value of the data**

• The dataset presented in this article can be used to investigate seed quality (seed germination rate, seed size and weight) of 16 wheat genotypes.
• The dataset represented here can be used to determine the solution concentrations of sodium chloride (NaCl) and calcium chloride (CaCl_2_·2H_2_O) to create solutions of different SAR.
• The dataset can be used to measure seedling emergence force of wheat or other crop genotypes.

## 1 Data

**Fig. 1:**
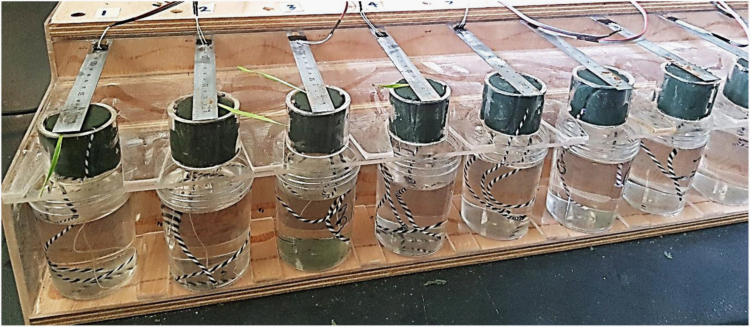
Experimental set up (arrangement 8 of 16 seedlings in each replicate) used to measure seedling emergence force.

**Fig. 2:**
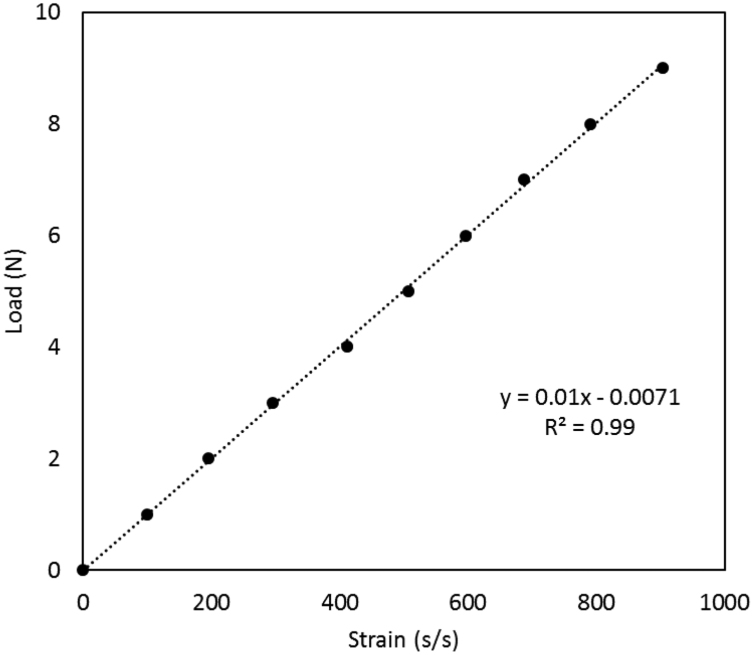
Relationship between the load (emergence force) and strain measured using the strain gauges in the experimental set up illustrated in Fig. 1.

This paper represents the dataset which have been used to investigate seed quality and germination rate of 16 wheat genotypes. Seed width and length was measured of these genotypes using slide calipers (Table 1) from 50 replicates of each genotype. The numbers inside the brackets in Table 1 represents the standard error between the genotypes. To create solutions of four different SAR (0, 10, 40 and 60) and four different *I* (25, 50, 300, 484 mmol L^−1^*)* different concentration of NaCl and CaCl_2_ were used which had been described in Table 2. A device was developed to measure the seedling emergence force of these 16 genotypes which had been shown in Fig. 1. This machine measured the deflection of a steel beam place on top of a foam when the seedling emerged from the foam and moved the beam. The deflection of the beam was measured using the stain gaze connected to the beam. The measured strain was converted to force using the calibration graph (Fig. 2) and seedling emergence force of each genotype was calculated.

**Table 1:** Seed germination (in non-limiting conditions), 100 seed weight, seed width and height of the 16 wheat genotypes. Data represent the mean ± the standard error for 5 replicates.

*Genotype*	*Total germination* (%)	*Thousand grain weight* (TGW, g)	*Seed width* (mm)	*Seed length* (mm)
*Aurora*	91 (±2)	46.1 (±1.6)	3.82 (±0.04)	7.71 (±0.11)
*Axe*	98 (±2)	37.5 (±2)	3.38 (±0.11)	6.52 (±0.09)
*Batavia*	96 (±3)	34.1 (±1.5)	3.58 (±0.16)	6.60 (±0.12)
*Baxter*	100 (±2)	37.0 (±2.2)	2.68 (±0.12)	6.75 (±0.04)
*Corack*	97 (±3)	32.0 (±2)	3.03 (±0.08)	7.33 (±0.21)
*Dharwar*	99 (±1)	40.1 (±2.1)	3.41 (±0.09)	7.01 (±0.22)
*Gregory*	99 (±1)	33.5 (±1.4)	3.01 (±0.11)	6.30 (±0.19)
*Impala*	98 (±2)	31.5 (±1.9)	3.03 (±0.08)	5.50 (±0.07)
*Krichauff*	100 (±1)	32.1 (±2.2)	3.10 (±0.12)	6.00 (±0.21)
*Lancer*	100 (±1)	35.0 (±1)	4.10 (±0.11)	7.30 (±0.25)
*Mitch*	100 (±1)	33.5 (±1.1)	3.20 (±0.12)	6.78 (±0.09)
*Seri*	99 (±1)	37.5 (±1.4)	4.01 (±0.21)	6.80 (±0.34)
*Spitfire*	100 (±1)	36.5 (±1.9)	3.30 (±0.23)	6.35 (±0.26)
*Ventura*	100 (±1)	36.0 (±1.3)	3.30 (±0.11)	6.66 (±0.31)
*Viking*	100 (±1)	37.0 (±2)	3.15 (±0.21)	6.41 (±0.14)
*Wyalkatchem*	99 (±2)	31.5 (±2.1)	3.15 (±0.27)	7.12 (±0.24)

**Table 2:** Treatments used for Experiment 2 in which the effects of SAR and *I* on germination and seedling emergence force were investigated for wheat seedling.

*Treatment*	*SAR* (mmol_c_ L^−1^)^1/2^	*I* (mmol L^−1^)	*NaCl* (g L^−1^)	*CaCl*_*2*_*·2H*_*2*_*O* (g L^−1^)
*1 (Control)*	0	25	0	1.22
*2*	10	25	0.97	0.41
*3*	40	25	1.39	0.05
*4*	60	25	1.42	0.02
*5 (Control)*	0	50	0	2.45
*6*	10	50	1.59	1.11
*7*	40	50	2.67	0.19
*8*	60	50	2.78	0.09
*9 (Control)*	0	300	0	9.80
*10*	10	300	3.87	6.53
*11*	40	300	8.99	2.21
*12*	60	300	10.12	1.24
*13 (Control)*	0	484	0	23.7
*14*	10	484	6.46	18.2
*15*	40	484	17.81	8.67
*16*	60	484	21.45	5.58

## 2 Experimental design, materials and methods

Seed samples of wheat genotypes were collected from a harvest site at the Queensland Government research farm in Kingsthorpe, Queensland, Australia (27.52 °S, 151.79 °E). After harvest, seeds were stored in cold temperature (7 °C), and before one week of seed germination test, width and length measurement, seeds were warmed to 22 °C. For each variety, the thousand-grain weight (TGW, Table 1) was estimated from the weight of 200 seeds from five replicate samples. Using digital slide calipers, the length and width of the seeds were measured for 50 replicate seeds of each genotype (Table 1).

Seedling emergence force of these 16 genotypes were measured in 16 treatment solutions, consisting of four ionic strength (*I)* values [25, 50, 300 and 484 mmol L^−^^1,^ corresponding to EC values of 0.16, 0.31, 1.24 and 3.0 dS m^−1^] and four SAR values (0, 10, 40, and 60) (Table 2). A mechanical device was developed (Fig. 1) to record the force exerted by the seed coleoptile. The device consisted of a stainless steel beam of 0.4 mm thickness and a width of 20 mm suspended above a seed. A strain gauge was attached to the beam to measure the displacement of the beam over time. The wheat seed was placed in a piece of foam that was held underneath the stainless steel beam. A 100 mL container of 1 mM CaCl_2_ [ionic strength *(I)* of 0.003 M L^−1^] was placed underneath the foam and cotton strings coming out from the foam were placed in the solution. The seeds germinated inside the foam and grew for total 14 d. When the seedling emerged, it pushed and moved the beam upwards. The attached strain gauge measured the movement of the beam from which the emergence force of the seedling was calculated. The strain gauge was connected to a data logger (*Quantum X data acquisition system DA, MX840B, HBM, Germany)*. Measured strain was converted to emergence force (N) using a calibration with strain measured at loads ranging from 0 to 9 N at 1.0 N intervals (Fig. 2).
